# A System of Practical Surgery

**Published:** 1843-01

**Authors:** 


### ART. XVII.

*A System of Practical Surgery.* By WILLIAM FERGUSSON, F.R.S.E., Professor of Surgery in King's College, London; Surgeon to King's College Hospital, &c. &c. With 246 Illustrations by BAGG.—*London,* 1842. 8vo, pp. 596.

THE object and nature of this volume are thus described by the author:

"THE present work has not been produced to compete with any already before the profession: the arrangement, the manner in which the subjects have been treated, and the illustrations, are all different from any of the kind in the English language. It is not intended to be placed in comparison with the elementary systems of Mr. Samuel Cooper, Mr. John Burns, Mr. Liston, Mr. Syme, Mr. Lizars, and that excellent epitome by Mr. Druitt. It may with more propriety be likened to the 'Operative Surgery' of Sir C. Bell, and that of Mr. Averill, both excellent in their day, or the more modern production of Mr. Hargrave, and the 'Practical Surgery' of Mr. Liston, which are so well known, and so justly appreciated. ...... There are subjects treated of in this volume, however, which none of these gentlemen have noticed, and the author is sufficiently sanguine to entertain the idea, that this work may, in some degree, assume that relative position in British surgery, which the classical volumes of Velpeau and Malgaigne occupy on the continent." (Pref. ix.)

Having in this extract given the author's own account of the work, we shall briefly state the nature of its contents, and try to enable our readers to appreciate how far Mr. Fergusson has succeeded in attaining his object.

The first section of the work treats of the "elements of practical surgery," and has been properly introduced with a view to avoid frequent repetitions elsewhere, which could otherwise have been scarcely avoided. Under the term practical surgery Mr. Fergusson says he includes "the symptoms of disease and injury, the principles and objects of treatment, and such medicinal means of cure as seem to me to belong to the province of surgery." (p. 2.) The remainder of the work is in fact a treatise on operative surgery: it is divided into four sections, in which the superior extremity, the inferior extremity, the head and neck, and the chest, abdomen, and pelvis, are respectively treated of under the several heads of dissection, dislocations, fractures, and the various operations that are liable to be performed in each particular region. It is of course impossible that we should give an analysis of a book thus embracing nearly the entire province of surgery; but we shall endeavour to estimate its general character, and to convey a fair and just idea of Mr. Fergusson's matter and manner.

And first we must observe that we have formed a decidedly favorable opinion of Mr. Fergusson's work; we have been surprised that so many important matters could have been so well stated in so small a space; but we have at the same time been struck with some imperfections and deficiencies, which we deem it our duty to notice.

The chief blemish of the work is, that some points are so superficially treated that the information conveyed respecting them is very imperfect, we might say useless for practical purposes. This observation applies more particularly to the section "elements of practical surgery." So much of it as relates to what is commonly termed "lesser surgery" is full and satisfactory, but some of the subsequent articles are superficial and meager: in some instances important practical points are mentioned but not discussed, the proposition is enunciated but there is no attempt at its solution, no rules laid down for its practical application, and in other instances the symptoms of disease are disposed of with similar brevity.

Thus, for example, in discussing the treatment of inflammation, after the ordinary antiphlogistic measures are enumerated, we are told:

"Besides these measures, which may be said to constitute the most important parts of the constitutional treatment of inflammation, there are others, which often are of the last consequence, such as opiates, to allay irritation, and stimulants, to rouse the depressed and sinking powers of life. The exhibition of camphor, ammonia, and wine may prove of the utmost value in certain cases, which form, as it were, the exceptions to the general practice of constitutional depletion, which is so universally admitted as being the proper course to pursue in the treatment of this disease." (p. 52.)

These observations would constitute an excellent introduction to an account of the characters and symptoms of the class of cases referred to, but there is literally not one word on that point; we are merely informed that such cases do exist. In speaking of the signs of suppuration, the fallaciousness and insufficiency of the general symptoms as indicative of its occurrence, is clearly and judiciously pointed out, the importance of fluctuation as a diagnostic sign is then mentioned, and it is said (p. 61), "the feeling of fluctuation is well understood by the person who has once placed the fingers of one hand over the collection of matter, and tapped gently on the swelling with those of the other." We could have wished for more detail respecting the important practical points of the mode of detecting fluctuation, and of the deceptions both positive and negative that may so readily occur in attempting to do so; and we may further remark that œdema, occasionally so important an indication of the formation of deep-seated matter, is not hinted at.

In the chapter on erysipelas (p. 87, et seq.) there is, we may literally say, not one single word respecting the symptoms of the affection; the disease is *assumed* to be known. Again in the account of the diagnosis of aneurism (p. 106), the ordinary symptoms are well and clearly detailed, and it is then stated that some of those "symptoms are occasionally indistinct, and others not altogether to be relied on." This is very true; but we see little use in mentioning the fact without appending an appreciation of the comparative value of the symptoms, and an account of the methods of endeavouring to establish a diagnosis in an obscure case, which is not done.

The portion of the work in which the affections incidental to each region are described is nearly free, yet not absolutely exempt, from faults of the kind we are now noticing. Thus there is positively no account given of the symptoms of dislocation of the radius either backwards or forwards, further than that of the latter accident we are told "it seems to produce little inconvenience, saving a slight diminution of flexion and extension, more particularly of the former." (p. 157.) It is needless to say that the symptoms of these accidents are characteristic enough, and that they cause much pain and inconvenience at and for some time after the period of their occurrence. As to the treatment of these accidents, Mr. Fergusson says "where either of these injuries is detected at first, the head of the bone should be drawn by extending or bending the elbow, or perhaps thrust at once into its proper position." It would have been shorter, and practically just as useful, to have said, "These dislocations when detected should be reduced." They usually are easily replaced; but we know from experience that their reduction is sometimes difficult enough unless the forearm, while being flexed, is also simultaneously pronated or supinated, according as the displacement is forwards or backwards. With respect to dislocations of the forearm backwards, we are simply told "the nature of the accident will be readily made out by any one acquainted with the anatomy of the joint." (p. 157.) Again there is no hint of the peculiar symptoms of fracture of the lower end of the radius, of the imperfections it may produce in the motion of the hand if not successfully treated, or of the difficulties in the way of so treating it. (p. 175.) Such omissions have struck us the more, as the plan of the work includes "the symptoms of disease and injury," (p. 2,) and as we find some points of secondary interest, because of very rare occurrence, discussed at some length, e. g.: the propriety of operating for aneurism while a female is pregnant. We must not, however, be understood as implying that superficial and defective description of the symptoms and treatment of disease and injury are characteristics of Mr. Fergusson's work; on the contrary, the instances of such defects which we have noticed, with some others that we might notice, constitute the comparatively few exceptions; and perhaps on that very account have the more forcibly attracted our attention.

We must also object to the way in which several both of the minor and greater questions of surgery are dogmatically decided, without any discussion, without any reasons assigned for the conclusion at which the author arrives. Thus at p. 96 we find it stated that "inflammation is the inevitable consequence of all wounds:" but surely this is at the least an important unsettled question; the reasoning and facts adduced by Dr. Macartney are not to be thus cavalierly disposed of; and the phenomena of subcutaneous wounds, which are discussed in this very chapter, should make us hesitate in arriving at such a dogmatic conclusion. At p. 155 dislocations of the wrist, without any accompanying fracture, are unreservedly admitted as of common occurrence, and no hint is given of the difference of opinion existing on this point among surgeons of the greatest experience. At pp. 92 and 318 the danger of operative interference with veins is decidedly underrated. There is indeed some discussion on this point, so that perhaps a notice of Mr. Fergusson's opinion respecting it is here misplaced; we may, however, observe that Mr. Fergusson regards the prevalent notions respecting the danger of certain operations on the veins as decidedly exaggerated, an opinion partly formed on the alleged success of some French practitioners in these operations. Almost while we are writing these lines we have read of two fatal cases of phlebitis following the application of needles for varix of the lower extremity, which occurred in the practice of Roux, and are published in the Gazette des Hôpitaux, Nov. 5th. With respect to the great question of the occasional occurrence of death from the entrance of air into a vein during the performance of an operation in the vicinity of the neck, Mr. Fergusson says "a few globules which may accidentally be admitted cannot do that harm which some have imagined." No one, we believe, ever imagined that the introduction of a *few globules* could do much harm, and we do not approve of so brief and inconclusive a decision of so important a question, the more especially as we believe it to be positively demonstrated that death has in *many* instances occurred from the accident in question. And here we may be allowed to mention that another example of this lamentable event has been communicated by M. Gomé to the Academy of Medicine of France so lately as the 3d of November.

Having now exhausted every topic of blame, we gladly apply ourselves to the more grateful task of laudatory comment; and as we have not hesitated to find fault when we considered it called for, our praise we trust will carry with it the greater weight.

The surgical anatomy of each region is described in a manner at once clear, compendious, and well calculated, as the author desires, to "encourage ocular inspection" rather "than to facilitate the prevalent, baneful, and schoolboy system of acquiring names from printed lessons." (Preface, p. viii.) The plan of this portion of the work has, in our opinion, been admirably conceived and as well executed.

We extract the following observations respecting the diagnosis of malignant tumours as an average specimen of Mr. Fergusson's semeiological and diagnostic details, and because the fact that it is but an average specimen sufficiently shows that our former strictures apply to but a comparatively small portion of the book :

"The symptoms do not always indicate the character now under consideration (malignancy). For instance, pain is not a test of malignancy ; for the most painful of all, perhaps, is the painful subcutaneous tubercle, which, however, is not malignant ; whilst, on the other hand, medullary sarcoma, one of the most malignant of all growths, is not characterized by remarkable pain. The peculiar pricking, lancinating pain of scirrhus, is often a good test of the disease, but frequently such tumours are met with in which this symptom is by no means conspicuous. Rapid growth is probably a more certain test of malignancy, but many exceptions to this may be met with also. I am most inclined to take as good criteria the apparent effect which tumours have upon the constitution, and the extent of their connexion with the neighbouring parts. If a patient, after a growth has existed for some time, gradually loses flesh, becomes pale and languid, has constant uneasiness if not pain in and around the part, I consider these good grounds for suspicion that the affection is malignant ; and if, in addition, the local disease has no distinct limits, (in other words, if the exact line of separation between the sound and affected parts cannot be made out,) if the tumour be in a manner fixed, if the skin over it does not appear healthy, however much it may be stretched, and does not glide over the swelling, and if the latter does not move readily on the subjacent parts ; if, added to these, the whole, or a great part of the thickness of a limb, or wherever the disease may be, seems to be more or less involved, there need be little doubt that the disease is malignant. Some objections to these general characters will at once be perceived ; as, for example, a tumour of bone cannot be moved like one of the soft parts ; and, in certain instances, there are additional characters which indicate peculiar diseases, as the ulceration in the latter stages of scirrhus, or the bleeding growth in fungus hematodes." (pp. 120-1.)

The comparative appreciation of the flap and circular methods of amputation affords a fair example of the candid and judicious manner in which Mr. Fergusson discusses an important surgical question, especially when we consider that it is written by one who himself almost invariably adopts the flap operation.

"If rapidity of execution is to be taken as the test of superiority, then I imagine that the flap operation must be allowed the preference, but in the hands of a good surgeon the difference of time required for the efficient performance of either, seems to me of so little consequence, that such a calculation should not be taken into account . . . . . I cannot but think that the same hand which rapidly and safely completes the flap incision, would with almost equal facility, if equally well trained, accomplish the circular. . . . . A surgeon of the present day who takes more than from thirty seconds to three minutes (excepting under peculiar circumstances) for the performance of an amputation, whether flap or circular, ought not in my opinion to be taken as an authority on the subject. I would therefore set aside the question of time in such an argument as that to which I now allude. The comparative extent of cut surfaces in the respective operations seems to me of trifling import; a few inches more or less, provided always that a good stump is left, will never in my opinion determine the issue of an amputation. The bleeding during such a proceeding is greatly, perhaps entirely, under the control of the surgeon. It has been asserted by Sir G. Ballingall and others, that the vessels retract more completely in the circular operation. . . . . It must be admitted, however, that if the bleeding is more copious in the flaps, it is easier to get at the deep part of the wound than in the hollow cone of the circular incision. . . . . It has been asserted in most controversies on this question, that in the circular operation the skin alone is left to cover the end of the bone, whereas in the flap method there is a cushion of muscular fibres preserved which afterwards protects that part, and lessens the chance of injury from pressure. The nature of the covering, however, depends greatly on the manner in which the operation is performed. . . . If the incisions are so managed as to leave a sloping surface from the edge of the divided bone to that of the skin, and if a sufficiency of soft parts be left, the covering of the bone may thus be as thick (as muscular) as by the flap operation. . . . Whether the operation has been by flap or by circular wound, the stumps are at last so much alike in certain parts of the body, that it is occasionally difficult after the lapse of years to say whether an amputation has been by one mode or the other: at all events, when such distinction can be drawn from the shape of the cicatrices, it is evident that the end of the bone is covered by much the same thickness of soft parts in one instance as in the other. . . . . Non-union, suppuration, and granulation, may happen from one operation as well as the other. . . . . I feel bound to state, that I have seen adhesion by the first intention follow as perfectly after the circular operation as after the flap; and, in addition, I have seen as good stumps from the one as the other." (pp. 126-8).

In describing the particular operations, Mr. Fergusson contents himself with detailing those methods which in each case he thinks deserve the preference; thus he does not describe the twenty or thirty different modes which have been proposed for amputation at the shoulder-joint. He inculcates a study of the anatomy of the parts rather than of the detail of printed opinions:

"I do not mean by these remarks to discourage the study of the opinions and proceedings of many who are justly considered our best authorities on such matters; but, as it is not within the compass of this work to describe, or allude to all that has been said or done in surgery, or to point out the trifling shades of difference in the practice of twenty or thirty individuals—each of whom, be it remarked, is an authority perhaps equal to any other,—I shall rest satisfied with describing two methods which I have myself resorted to on the living body, after having given to most of the others a fair consideration and trial on the subject." (p. 230.)

The various methods recommended by the author for the performance of every operation of any importance, are described with great clearness and precision, selected with great judgment, and most admirably illustrated by woodcuts that in design and execution leave nothing to be wished for, and which, instead of being collected at the end of the volume, are intercalated with the text, so as to most conveniently aid the reader in following the descriptive details which they so well exemplify. Mr. Fergusson, we may here observe, makes light of many rules to which very undue importance have been attached in the performance of certain operations; as an example of this we may refer to his observations on amputation of the leg, with reference to the supposed necessity of the surgeon standing on a certain side of the limb, and sawing the fibula before the tibia is divided. (p. 354-5.) We fully concur in the justice of these and other similar observations, and may add that our author has not gone too far in this direction, as he carefully notices those rules that are essential for the convenient and safe achievement of almost every important operation. A few extracts will best show how far this opinion is well founded.

In describing excision of the elbow-joint, Mr. Fergusson says:

"The main object in such a proceeding is to remove all the diseased portions of bone, and this ought to be accomplished with as little injury to the surrounding parts as circumstances will permit. By laying open certain sinuses, which may exist in some instances, sufficient room may be obtained, but in general it will be better to make either a crucial incision + or one in the form of H. The posterior surface of the joint is evidently the most eligible part to make a free opening, the large artery and nerves, with the exception of the ulnar, all lying in front, being separated, too, from the bones by the brachialis anticus muscle. The operation is accordingly always done on the dorsal aspect. The patient may be either laid on a table with the face downwards, or be seated on a chair; the former position ensures greater steadiness on his part, and I have seen it most frequently preferred, but the latter I have myself selected, as I think the operator can so move the arm as to enable him to inspect the ends of the bones more accurately than whilst the patient is kept on his face. I believe however the position of the patient is of less moment than some seem to imagine; whichever attitude is fixed upon, the arm and forearm should be firmly held by an assistant, then if the extent of the disease is not supposed to be great, a crucial incision should be made directly over the olecranum; but if, on the contrary, a free exposure of the parts be deemed requisite, the other incision should be selected. On raising the flaps, which should consist of the entire thickness of the skin, as well as the condensed and infiltrated cellular texture underneath, the olecranum process will be laid bare; in cutting on its inner margin, the ulnar nerve must be carefully preserved from injury, which can be best done by carefully dissecting it out of its position behind the internal condyle at this stage of the operation, and holding it aside with a blunt hook during the future steps; the attachment of the triceps should next be divided, and the cutting forceps used to separate the olecranum from the ulna. The surgeon will now be enabled to appreciate the condition of the articular surfaces more clearly than heretofore; and will be guided in his future steps by the apparent extent of the disease. He may now, with the forceps, divide the remaining portion of the upper end of the ulna, the head of the radius, and whatever part of the humerus he may deem necessary. In the adult the saw may be necessary for the latter purpose, but in a young patient there is no difficulty in effecting this object with the instrument recommended, and occasionally the gouge may be of service in scooping away small spots of the carious surface which cannot be reached by either forceps or saw. . . . . . In some instances the lateral ligaments must be cut through, to allow the ends of the bones to be fairly turned out; and in all cases of extensive disease, this had better be done at once; in doing so, however, there is no necessity for exposing such an extent of their shafts as was formerly deemed requisite, as the saw may be used with perfect ease, without the presence of a broad spatula in front of the bone, as is recommended by some even in the present day; indeed, the method of removing the diseased portions of bone here described, seems to me to constitute a most important difference from that resorted to by Moreau, who, in separating the 'enlarged and rough' end of the humerus, doubtless went far beyond the actual disease, and thus made the wound unnecessarily extensive and severe......I hold it to be of the utmost consequence, in the performance of this operation, to distinguish between caries and that enlarged and hardened condition of the bone in the immediate vicinity of this disease, which, though altered in structure, may be allowed to remain, whilst, to ensure success, the former must be taken away......It most commonly appears that the caries is limited to the articular surfaces, or their immediate vicinity; and though large portions of the shafts of the bones have been occasionally removed with success, when the disease has necessitated such extensive wounds, a judicious advocate for excision would, in all probability, in such a case give a preference to amputation." (pp. 205-7.)

We had marked some other passages for quotation, but must abstain from extracting them, as we have already said enough to convey an adequate idea of the nature and execution of Mr. Fergusson's treatise, which ought to be in the hands of every surgeon. We must, however, make room for the following passage, in which an ingenious method was adopted for restoring the symmetry of the nose:

"A patient of mine, (a young gentleman who had been somewhat vain of his personal appearance previously, and not without good reason,) was left in the condition represented in fig. 206. The columna and the cartilaginous septum had been destroyed, and the vomer had separated by necrosis. Here I imagined that a modification of the proceeding of the Berlin professor might be advantageously resorted to, and accordingly proceeded thus. The patient being seated, the point of a small scalpel was introduced under the apex, and the alæ were separated from the parts underneath; next the knife was carried on each side between the skin and the bones, as far as the infra-orbital foramen, taking care not to interfere with the nerves, when by passing the point of my finger below the nose, I caused the latter organ to be as prominent as could be wished. I now pushed a couple of long silver needles, which had been prepared for the purpose, with round heads and steel points, across from one cheek to the other, having previously applied on each side a small piece of sole leather perforated with holes at a proper distance, then I cut off the steel points, and with tweezers so twisted the end of each needle as to cause the cheeks to come closer to each other, and thus render the nose prominent......thus by bringing the cheeks more into the mesial line, a new foundation, as it were, was given to the organ." (pp. 453-4.)

The operation, to judge from the woodcuts annexed, succeeded perfectly.

We may refer to the account of dislocation of the hip-joint, of hernia, of operations on the superior and inferior maxillæ, and on the great arteries in the neck, as a few out of many examples, showing what a mass of valuable information respecting some of the most important points in surgery, Mr. Fergusson has contrived to compress within very moderate limits. We must, however, conclude. It is scarcely necessary to say, that we deem Mr. Fergusson's work to be very valuable, and practically useful: any little imperfections we have noticed are far outweighed by its merits; and the present treatise cannot but enhance the reputation of its author as a judicious and experienced practitioner. The woodcuts are in Bagg's happiest manner, and are indeed beyond all praise.

